# Fish protein supplementation in older nursing home residents: a randomised, double-blind, pilot study

**DOI:** 10.1186/s40814-019-0421-x

**Published:** 2019-02-26

**Authors:** Aslaug Drotningsvik, Åge Oterhals, Ola Flesland, Ottar Nygård, Oddrun A. Gudbrandsen

**Affiliations:** 1 Dietary Protein Research Group, Department of Clinical Medicine, University of Bergen, PO Box 7804, N-5021 Bergen, Norway; 2 TripleNine Vedde AS, 6030 Langevåg, Norway; 3 Nofima, P.O. Box 1425, Oasen, 5828 Bergen, Norway; 4 Department of Clinical Science, University of Bergen, 5021 Bergen, Norway; 5 Department of Heart Disease, Haukeland University Hospital, Bergen, Norway

**Keywords:** Fish protein, Frail elderly, Nursing homes, Sarcopenia, Pilot study

## Abstract

**Background:**

Age-related loss of muscle mass and function is common in older adults, and studies investigating if dietary proteins may protect and possibly build lean body mass are needed. We assessed the feasibility of conducting a nutritional intervention study in older nursing home residents to investigate the effects of fish protein supplementation on markers of glucose metabolism and inflammation.

**Methods:**

This was a double-blind randomised controlled pilot study. Twenty-four nursing home residents, without major cognitive impairment, received a daily oral nutritional supplement containing 5.2 g of fish protein or placebo for 6 weeks. Anthropometric measurements were conducted at baseline. Participants were screened for nutritional risk using the Mini Nutritional Assessment and activities of daily living using the Barthel index and dietary intake was registered. Hand grip strength was measured and fasting blood samples collected at baseline and endpoint.

**Results:**

Compliance was high and dropout was low, but participant recruitment was challenging. Serum concentrations of monocyte chemoattractant protein-1 decreased, and C-reactive protein increased in the intervention group compared to control, with no changes in markers of glucose metabolism between groups.

**Conclusion:**

Conducting a nutritional intervention using fish protein supplementation in older nursing home residents is feasible but should be conducted as a multi-centre study to account for the low recruitment rate observed in the present study. A full-scale study is needed to gain more knowledge on the potential effects of fish proteins on markers of glucose metabolism and inflammation in relation to the age-related loss of muscle mass and function.

**Trial registration:**

ClinicalTrials.gov NCT03529344 18.05.2018 (retrospectively registered)

## Background

Age-related loss of muscle mass and function is common in the older adult population and can lead to disability, loss of independence and increased risk of death [1, 2]. Insulin resistance and inflammation are associated with low skeletal muscle mass in the older adult population [3–6], and both are considered major factors involved in the development of age-related loss of muscle mass and function [7].

The role of nutrition in the prevention and treatment of muscle loss in the older adult population is not fully understood, and there is a need for studies investigating if different macronutrients, especially proteins, may protect and possibly build lean body mass [1]. In the nursing home setting, the prevalence of muscle loss and weakness is reported to be high [8, 9], and optimal nutritional treatment may be one strategy to prevent a decline in muscle function. To date, few nutritional intervention studies target nursing home residents, and more research is needed to optimise nutritional support for this population.

Currently, the older adult population is recommended a higher protein intake relative to body weight than the young adult population [10]. With advancing age, the balance between muscle protein synthesis and breakdown is altered and research indicates a lower muscle protein synthesis in the older compared to younger adults [11]. The lower muscle protein synthesis is suggested to be a consequence of an anabolic resistance in the ageing muscle, where the muscle is less sensitive to anabolic stimulants such as insulin, amino acids and physical activity [12]. Nutritional interventions have focused on providing optimal amounts of nutrients including proteins, but little is known about whether altering the type of dietary proteins could beneficially affect the development of age-related loss of muscle mass and function in older adults. In rats, fish protein feeding has been shown to beneficially affect fasting [13, 14] and postprandial [15–17] glucose concentrations. Clinical studies show lower fasting and postprandial glucose concentrations in overweight or obese adults after fish protein intake [18] and improved insulin sensitivity in insulin-resistant adults after lean fish intake [19]. Also, some studies have shown a potential for fish proteins and lean fish intake to beneficially affect chronic low-grade inflammation [20, 21]. Fish proteins could therefore have a beneficial effect on muscle preservation.

Blue whiting (*Micromesistius poutassou*) is a small pelagic fish species used primarily to produce fish meal for the aquaculture industry [22]. Proteins from small fishes such as blue whiting constitute an unutilized source of high-quality proteins that could be used for human consumption. We have previously shown that blue whiting protein feeding led to lower circulating cholesterol [23] and beneficially affected hypertension (unpublished data) in obese Zucker fa/fa rats, indicating that in addition to being a source of protein, blue whiting proteins may exert specific beneficial metabolic effects. Investigating potential beneficial effects of fish proteins on parameters related to reduced muscle function and mass such as glucose metabolism and inflammatory status are of interest and could lead to improved nutritional treatment for older adults.

The aim of the present study was to assess the feasibility of conducting a nutritional intervention in older nursing home residents in Norway where effects of a daily supplementation of fish protein on parameters related to glucose metabolism and inflammation were investigated.

## Methods

### Study design

The study was designed as a double-blind, randomised, controlled pilot study investigating the effects of a daily supplementation of fish protein on markers of glucose metabolism and inflammation in older nursing home residents. The study had two arms: one intervention arm and one control arm. Participants consumed 5.2 g of blue whiting protein hydrolysate or placebo daily for 6 weeks. A total of 24 participants were included in the study, and 21 completed the intervention. Participants were stratified according to sex, age and weight before randomisation to one of the two experimental groups.

Eligibility criteria were changed after pilot trial commencement. Originally, we aimed at recruiting older nursing home residents who met the criteria for sarcopenia as defined by the European Working Group on Sarcopenia in Older People (EWGSOP) [1]. During the first week of participant recruitment, we decided to abandon the sarcopenia criteria due to the low number of eligible participants. Thus, all participants in the present study followed the changed criteria which included older nursing home residents in Bergen, Norway who were without major cognitive impairment and were willing to participate.

### Participants

Participants were recruited during two time periods: September to October 2016 and September to November 2017, and the study was conducted in October and November 2016 and in October, November and December 2017. All nursing homes in Bergen were invited to participate in the study either by e-mail or phone, and of the 30 nursing homes contacted, only 6 was interested in study participation. The main reasons for not entering the study was a low number of eligible participants and that study participation was considered too demanding for the nursing home staff. Participant inclusion criteria were age 60 years or older, living in a nursing home and willingness to take a daily oral protein supplement during the intervention period. Exclusion criteria were life expectancy < 6 months, weight loss > 5 kg during the last 3 months, major cognitive impairment and allergy towards fish or other major food groups. A list of eligible participants was provided by the nursing home staff. Eligible participants received information about the study, and those interested in entering the study were visited on a subsequent day for collection of written informed consent and baseline examinations. The study was conducted in accordance with the guidelines laid down by the Declaration of Helsinki and was approved by the Regional Ethics Committee of Western Norway (approval no.: 2015/75). The study was conducted in collaboration with the Agency for Nursing Homes, Department of Health and Care in Bergen Municipality. All participants gave their written informed consent and were explained that they were free to withdraw from the study at any timepoint during the intervention without having to give a reason and without negative consequences for future care. The study is registered at ClinicalTrials.gov (NCT03529344).

Health professionals performing blood sampling, anthropometric measurements and measurement of hand grip strength, and personnel conducting the laboratory analyses were blinded. All the data were analysed anonymously.

### Intervention

Participants received a daily oral supplement of either fish protein or placebo for 6 weeks. Before study-start, various ways of masking the fish flavour were tested (such as chocolate powder and different soft drink flavours), and we found that the taste and smell of the fish protein powder were efficiently masked by a lemon/lime or strawberry/kiwi flavoured soft drink. The supplement was prepared by the nursing home staff as a drink where 5.2 g of blue whiting protein hydrolysate was added to a non-caloric soft drink (Fun light, Orkla Food Norge, Trollåsen, Norway) in the intervention group. The control group received the same non-caloric soft drink without protein supplement. Fish protein was provided to the nursing homes as a powder, in pre-packaged portions containing 5.2 g protein. The drink was served in a cup with lid and straw. Participants could choose between the two juice flavours, lemon/lime and strawberry/kiwi. Intake of the drink was registered daily by the nursing home staff. All participants were instructed to maintain their normal eating habits during the intervention period. Participants were visited regularly by study personnel to ensure that the intervention did not cause any discomfort such as gastrointestinal pain, nausea or reduced appetite.

### Preparation of fish protein hydrolysate from blue whiting

The blue whiting was unloaded at TripleNine Vedde AS fish meal and fish oil factory, and the blue whiting protein hydrolysate was produced by SINTEF Ocean (Trondheim, Norway). In brief, the fish was grinded and mixed with water 1:1, heated to 55 °C and added 0.1% Protamex® (NovoZyme AS, Bagsværd, Denmark) (residence time 60 min). The hydrolysate was separated by a three-phase decanter centrifuge (Flottweg, Vilsbiburg, Germany), and the obtained liquid phase was membrane filtered by Membranteknikk AS (Flekkefjord, Norway); first microfiltration (pore size 0.1 μm, Tami, Nyons, France), then nanofiltration (retention size > 200–400 Da, CSM, Seoul, South Korea). The hydrolysate was spray dried by Nofima (Bergen, Norway) using a P-6.3 spray dryer (Niro, Sjøborg, Denmark) with an inlet temperature of 200 °C and an outlet temperature of 94 °C.

### Anthropometric measurements

Mid-upper arm circumference, calf circumference, and biceps and triceps skinfold thicknesses were measured at baseline. The measurements were conducted using the International Standards for Anthropometric Measurements [24] with minor adjustments due to impaired mobility of the participants. Subjects were in a sitting position, with arms hanging by the side to measure arm circumference and skinfold thicknesses. The relaxed arm circumference was measured at the mid acromiale radiale site using an anthropometrical tape (Cescorf Equipamentos para Esporte Ltda. Porto Alegre, Brasil), skinfold thickness was measured at the triceps and biceps skinfold sites using the Harpenden skinfold calliper (Baty International Ltd., West Sussex, UK). Calf circumference was measured when subjects were sitting at the site of maximal girth using Cescorf anthropometric tape. Body weight was recorded at baseline and endpoint using Seca 877 for mobile participants or by the nursing home staff using the scales at the institutions for participants with reduced mobility. Standing height of the participants could not be measured as most participants had impaired mobility. All anthropometric measurements were performed by the same assessor.

### Evaluation of hand grip strength

Hand grip strength was measured using the Baseline dynamometer (Fabrication Enterprises, NY, USA) at baseline and endpoint following the instructions from the Southampton grip-strength measurement protocol [25]. Hand grip strength measurements were performed by the same assessor at both visits.

### Collection of serum samples

Blood samples were collected at baseline and endpoint after an overnight fast. Participants were instructed to not eat or drink anything except water after 22.00 h the previous day. When considered medically safe, medications were taken after blood sampling. Blood samples were collected in BD Vacutainer SST II Advance gel tubes (Becton, Dickinson and Company) for isolation of serum. Blood samples were centrifuged 30–40 min after collection at the nursing homes, before transportation to the storage facility. Serum samples were stored at − 80 °C until analysis.

### Screening of nutritional risk, activities of daily living and registration of medications and medical diagnoses

Participants were screened for nutritional risk using the Mini Nutritional Assessment form (MNA) and were evaluated for their activities of daily living (ADL) using the Barthel ADL index [26]. MNA and ADL screenings were performed once during the study period by the nursing home staff familiar with the participants. Information about the participants’ medications and medical diagnoses were obtained from the electronic patient journal used by the nursing homes.

### Estimation of energy and protein intake

Dietary intake was registered for 24 h by the nursing home staff once during the intervention period. The dietary records were used to estimate participants’ intake of energy and proteins using the dietary assessment software Dietist XP version 3.2, with the Norwegian dietary composition table (Kost och Näringsdata, Stockholm AB, Sweden, http://www.kostdata.se/nb).

### Serum analyses

Serum concentrations of fructosamine (reflecting the glycaemic level over the last 2–3 weeks), glucose and C-reactive protein (CRP) were analysed on the Cobas c111 system (Roche Diagnostics GmbH, Marburg, Germany) using the FRA (Fructosamine) kit for Cobas c systems from Roche, and the GLUC2 (Glucose) kit and CRPHS (cardiac C-reactive protein high sensitive) kit for the Cobas c111 system from Roche. Serum concentrations of insulin, monocyte chemoattractant protein-1(MCP-1/CCL2) and interleukin-6 were analysed using the EIA-2935, EIA-4857 and EIA-4640 ELISA kits from DRG Instruments, GmbH, Marmburg, Germany. The concentration of serum interleukin-6 was below the detection limit for 8 of the 21 participants at either baseline and/or endpoint, and therefore, no statistical testing was conducted.

### Analyses of amino acid composition in the blue whiting protein hydrolysate

Analysis of amino acid composition in the blue whiting protein hydrolysate was conducted by Nofima BioLab (Bergen, Norway) using the methods by Cohen et al. [27] and Miller et al. [28]. The daily dosage of indispensable amino acids from the blue whiting protein hydrolysate is presented in Table 1. Total protein content was calculated as the sum of amino acids.

**Table 1:** Daily dosage of indispensable amino acids from the blue whiting protein hydrolysate supplement

Amino acids, mg/day	Blue whiting protein hydrolysate
Histidine	93.0
Isoleucine	198.4
Leucine	378.2
Lysine	477.4
Methionine	148.8
Phenylalanine	167.4
Threonine	223.2
Tryptophan	42.2
Valine	223.2

### Outcome

The primary outcome was to evaluate the feasibility of conducting a nutritional intervention study using fish proteins in elderly nursing home residents. The feasibility was assessed by measuring participant compliance with the intake of the intervention drink and participant dropout. Recruitment potential from the nursing home population in Bergen Municipality and adherence to sample collection protocol were also assessed. Secondary outcomes were to examine the effects of the fish protein supplementation on markers of glucose metabolism and inflammation in serum.

### Evaluation of feasibility outcomes

To evaluate participant compliance, intake of the intervention drink was registered daily by the nursing home staff and reviewed by the study personnel. Participant dropout was registered and completion rate was calculated. To assess recruitment potential from Bergen Municipality, the number of contacted and participating nursing homes was registered. Adherence to the sample collection protocol was registered.

### Sample size

The primary endpoint of this study was to assess the feasibility of conducting a nutritional intervention using fish proteins in elderly nursing home residents, and therefore, a formal sample size calculation was not performed. To evaluate the feasibility of the present study, we considered it necessary that 20 participants (about 10 per group) completed the intervention period. Older nursing home residents make up a heterogeneous study population with a wide array of diseases and large variations in health and functional status; therefore, we expected a high dropout rate in the present study and aimed at recruiting 30 participants per group.

### Statistical analyses

Statistical analyses were conducted using SPSS Statistics version 25 (SPSS, Inc., IBM Company, Armonk, NY, USA). All variables were evaluated for normality using the Shapiro-Wilks test, Q-Q plots and histograms. All continuous variables were analysed using parametric tests and non-normally distributed data were log-transformed before statistical testing. Changes within groups from baseline to endpoint were tested using paired samples *t* test, and independent samples *t* test was used to compare changes over time between the intervention group and control group. Pearson’s chi-squared test was used to compare categorical data. Level of significance was set to *p* < 0.05.

## Results

### Participant recruitment, compliance and characteristics

**Fig. 1:**
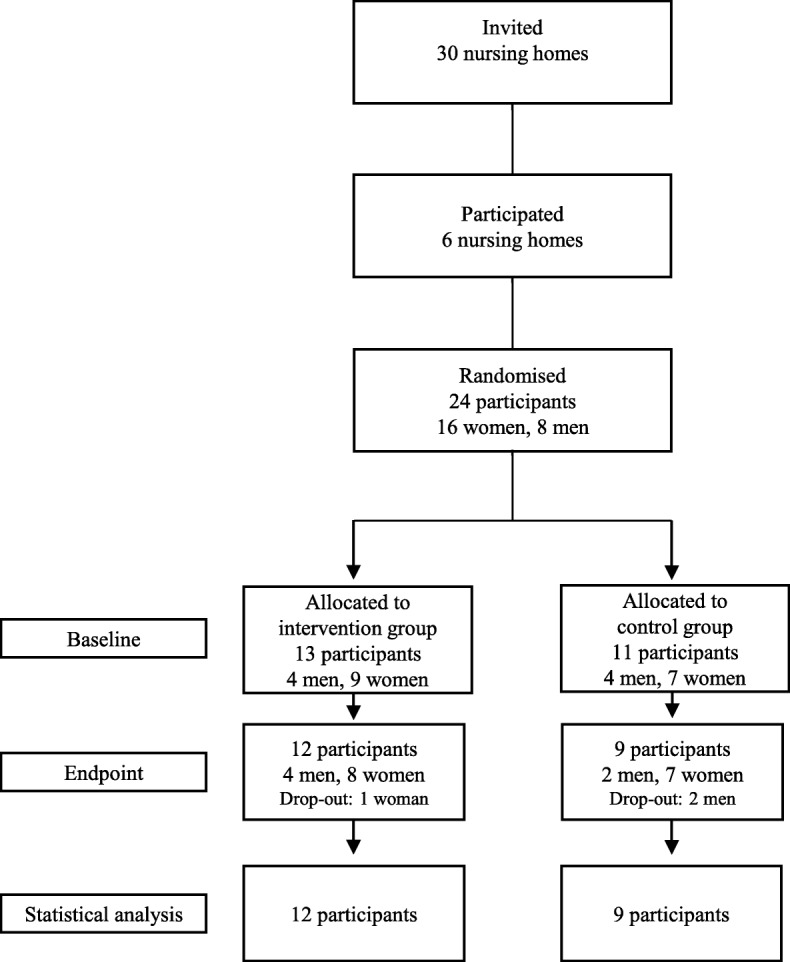
Flow diagram of study participants

Thirty nursing homes were invited to participate in the study and of these 6 nursing homes entered the study. From the 6 nursing homes, 24 participants (16 women, 8 men) were randomised to either intervention group or control group, and 21 participants completed the study. The participant completion rate of 88% shows that study conduction was feasible. Two participants, both from the control group, withdrew from the study and one participant in the intervention group was not able to complete the endpoint visit due to a sudden illness. The flow of participants is presented in Fig. 1. Participants were between 68 and 96 years old. Gender, age, upper arm circumference, calf circumference, biceps and triceps skinfold thicknesses, ADL and MNA scores, number of diagnoses and prescribed medications, energy intake and protein intake were similar between groups (Table 2**)**. Intake of the intervention drink was registered by the nursing home staff and showed good compliance by all participants.

**Table 2:** Participant characteristics at baseline

	Intervention group	Control group
	Mean (SD)	Mean (SD)
Women/men	8/4	7/2
Age, years	84 (8)	87 (5)
Upper arm circumference, cm	27.9 (3.2)	27.6 (3.8)
Calf circumference, cm	34.6 (2.5)	33.3 (5.9)
Biceps skinfold thickness, mm	8.5 (3.9)	8.2 (3.2)
Triceps skinfold thickness, mm	15.3 (5.2)	16.1 (4.7)
ADL score	14 (5)	12 (4)
MNA score	12 (1)	12 (2)
Number of diagnoses	5 (4)	4 (1)
Number of medications	10 (7)	9 (2)
Protein intake, g/kg BW/day	0.98 (0.30)	0.82 (0.17)
Energy intake, kcal/kg BW/day	22 (5)	23 (3)
Body weight, kg	69.4 (12.6)	63.7 (14.0)
Hand grip strength, kg	18 (8)	16 (11)

Data is presented as mean and standard deviation with *n* = 12 in the intervention group and *n* = 9 in the control group. Differences between groups were compared using independent samples *t* test; Pearson’s chi-squared test was used to compare categorical data between groups. *p* value < 0.05 was considered significant. No significant differences between groups were observed. *ADL* activities of daily living; *MNA* mini nutritional assessment; *BW* body weight

### Weight and hand grip strength

Weight and hand grip strength were similar between groups at baseline (Table 2) and did not change from baseline to endpoint within or between the two groups (data not presented). In the total study population, baseline measurements of hand grip strength show that 5 of the men and 12 of the women were categorised as having low muscle strength defined by the EWGSOP (< 20 kg for women, < 30 kg for men) [1].

### Analyses in serum

Blood samples from all participants were successfully collected and centrifuged at the nursing homes in accordance with the sample collection protocol. At baseline, no significant differences were observed between the intervention group and control group for serum concentrations of MCP-1, CRP, glucose, insulin and fructosamine. Serum concentration of the inflammatory marker MCP-1 was significantly reduced (*p* = 0.0064) while serum concentration of CRP was significantly increased (*p* = 0.040) from baseline to endpoint in the intervention group when compared to the control group (Table 3). Serum concentrations of glucose, insulin and fructosamine were not affected by the intervention.

**Table 3:** Serum concentration of markers of inflammation and glucose metabolism

	Baseline	Endpoint	Mean difference in change between groups^a^
	Mean (SD)	Mean (SD)	Mean (95% CI)
MCP-1, pg/ml			
Intervention group	178 (70)	159 (63)	− 80 (− 146, − 13)*
Control group	364 (324)	425 (401)	
CRP, mg/l			
Intervention group	3.77 (7.68)	6.29 (8.91)	0.77 (− 6.83, 8.37)*
Control group	8.71 (11.50)	10.46 (17.14)	
Glucose, mmol/l			
Intervention group	5.82 (1.00)	5.77 (1.14)	− 0.02 (− 0.41, 0.38)
Control group	5.59 (0.62)	5.56 (0.62)	
Insulin, ng/ml			
Intervention group	0.36 (0.13)	0.34 (0.12)	− 0.09 (− 0.23,0.04)
Control group	0.51 (0.42)	0.58 (0.61)	
Fructosamine, μmol/l			
Intervention group	280 (40)	280 (36)	2.86 (−13.74, 19.46)
Control group	259 (26)	256 (31)	

Data is presented as mean and standard deviation with *n* = 12 in the intervention group and *n* = 9 in the control group. For serum insulin concentrations, one participant in the intervention group used insulin and was therefore excluded; thus, results are presented for *n* = 11 in the intervention group and *n* = 9 in the control group. The groups were similar at baseline (independent samples *t* test). MCP-1, CRP and insulin were log-transformed prior to testing; within-group changes were tested using paired samples *t* test, and no significant changes within groups were observed. Within group changes were compared between groups using independent samples *t* test. *p* value < 0.05 was considered significant. *MCP-1* monocyte chemoattractant protein-1; *CRP* C-reactive protein; *CI* confidence interval

^a^Mean difference in change between groups shows change in control group minus change in intervention group

*Reflects significant differences in mean change between intervention group and control group

## Discussion

The present study is a pilot designed to assess the feasibility of conducting a nutritional intervention study in older nursing home residents to investigate the effects of fish protein supplementation on markers of glucose metabolism and inflammation. Recruitment of nursing homes and nursing home residents was a major challenge in the present study. However, the good participant compliance combined with the low dropout rate shows that conducting this nutritional intervention study in collaboration with the nursing home staff was feasible.

In the present study, we wanted to use measurements and cut-off values for muscle mass and muscle strength or muscle performance proposed by the EWGSOP to describe the participants’ muscle mass and function [1]. We chose to use only muscle strength measurements as a measure of muscle function because most of the participants had impaired mobility to an extent that conducting performance testing was considered unsafe when performed by only one study personnel. Initially, we planned to measure muscle mass by bioelectrical impedance analysis (BIA), but this was abandoned early in the study due to difficulties obtaining accurate height recordings. Instead, measurements of weight, skinfold thicknesses, and arm and leg circumferences were used to describe the participants, and these measurements were feasible and well accepted by the participants.

Nursing home residents are a multi-morbid group with high prevalence of cognitive impairment [29], and as those with major cognitive impairment were excluded, we expected that participant recruitment would be challenging. Initially, we aimed at recruiting a total of 60 participants for the present study, but the low number of interested nursing homes and the high percentage of residents with cognitive impairment and/or severe frailty markedly limited the number of eligible participants. To reach our minimum criteria of 10 participants per group, we had to recruit participants during two time periods (autumn of 2016 and 2017) and from six different nursing homes. Initially, only participants who met the criteria for sarcopenia set by the EWGSOP [1] could enter the study, but due to recruitment difficulties, we included all eligible and interested nursing home residents. Study completion rate was 88%, with 21 of the 24 included participants completing the intervention. Our study population consists of frail older nursing home residents where low compliance and high dropout rate could be anticipated, but despite this, the compliance was high and the dropout was low, which shows that study participation was not too demanding for the participants.

Fasting serum samples were analysed to assess changes in glucose, insulin and fructosamine and in the inflammatory markers MCP-1 and CRP after fish protein intake since fish proteins and lean fish have been shown to beneficially affect glucose metabolism and low-grade chronic inflammation [13–18, 20, 21]. We found reduced concentrations of MCP-1, increased concentrations of CRP and no changes in markers of glucose metabolism in serum after fish protein supplementation. However, these results are uncertain due to the low sample size, and the effects of fish protein supplementation should be tested further in a study with higher statistical power.

The present study is a pilot study designed to assess the feasibility of conducting a nutritional intervention study on the effects of fish protein supplementation on glucose metabolism and inflammation in older nursing home residents. For a full-scale study, the following modifications should be implemented:

- A full-scale study should be conducted as a multi-centre study to expand the number of eligible candidates. A multi-centre study could also contribute to a more representative study population and reduce the impact of systematic errors from individual nursing homes.
- The inclusion of older adults with cognitive impairment should be considered in a full-scale study as this would be more representative for the nursing home population.
- BIA and muscle performance testing such as the Timed Up and Go Test should be conducted for identification of participants with low muscle mass and function, for such examinations a minimum of two trained personnel should be present.
- To obtain standardised BIA measurements and to accommodate participants with reduced mobility, weighing should be conducted on a medical chair scale and height, and BIA measurements should be conducted with participants lying in a supine position.
- The nutritional intervention should be served as a pre-packaged ready to serve drink to minimise the workload for the nursing home staff and to ensure blinding of the staff.
- Employing a designated study nurse for project management and logistics at each nursing home will make the administration of the intervention more manageable.

## Conclusion

To conclude, conducting an intervention study with fish protein supplementation in older nursing home residents without major cognitive impairment was feasible as shown by the high compliance and low dropout of the participants who entered the study. However, a major challenge in the present study was to recruit nursing homes and nursing home residents, and a full-scale study should be conducted as a multi-centre study to account for the low recruitment rate.

## Abbreviations

ADL: Activities of daily living
BIA: Bioelectrical impedance analysis
CRP: C-reactive protein
EWGSOP: European Working Group on Sarcopenia in Older People
MCP-1: Monocyte chemoattractant protein-1
MNA: Mini Nutritional Assessment form
